# Identifying New Potential Biomarkers in Adrenocortical Tumors Based on mRNA Expression Data Using Machine Learning

**DOI:** 10.3390/cancers13184671

**Published:** 2021-09-17

**Authors:** André Marquardt, Laura-Sophie Landwehr, Cristina L. Ronchi, Guido di Dalmazi, Anna Riester, Philip Kollmannsberger, Barbara Altieri, Martin Fassnacht, Silviu Sbiera

**Affiliations:** 1 Comprehensive Cancer Center Mainfranken, University Hospital, University of Würzburg, 97080 Würzburg, Germany; Fassnacht_M@ukw.de; 2 Institute of Pathology, University of Würzburg, 97080 Würzburg, Germany; 3 Interdisciplinary Center for Clinical Research, University Hospital Würzburg, 97080 Würzburg, Germany; 4 Bavarian Cancer Research Center (BZKF), 97080 Würzburg, Germany; 5 Division of Endocrinology and Diabetes, University Hospital, University of Würzburg, 97080 Würzburg, Germany; Landwehr_L@ukw.de (L.-S.L.); C.L.Ronchi@bham.ac.uk (C.L.R.); Altieri_B@ukw.de (B.A.); 6 Institute of Metabolism and Systems Research, University of Birmingham, Birmingham B15 2TT, UK; 7 Endocrinology Unit, Department of Medical and Surgical Sciences, University of Bologna, 40138 Bologna, Italy; guido.didalmazi@unibo.it; 8 Department of Endocrinology, Medizinische Klinik und Poliklinik IV, Ludwig-Maximilians-University, 80336 Munich, Germany; Anna.Riester@med.uni-muenchen.de; 9 Center for Computational and Theoretical Biology, University of Würzburg, 97074 Würzburg, Germany; Philip.Kollmannsberger@uni-wuerzburg.de

**Keywords:** adrenocortical carcinoma, in silico analysis, machine learning, bioinformatic clustering, biomarker prediction

## Abstract

**Simple Summary:**

Using a visual-based clustering method on the TCGA RNA sequencing data of a large adrenocortical carcinoma (ACC) cohort, we were able to classify these tumors in two distinct clusters largely overlapping with previously identified ones. As previously shown, the identified clusters also correlated with patient survival. Applying the visual clustering method to a second dataset also including benign adrenocortical samples additionally revealed that one of the ACC clusters is more closely located to the benign samples, providing a possible explanation for the better survival of this ACC cluster. Furthermore, the subsequent use of machine learning identified new possible biomarker genes with prognostic potential for this rare disease, that are significantly differentially expressed in the different survival clusters and should be further evaluated.

**Abstract:**

Adrenocortical carcinoma (ACC) is a rare disease, associated with poor survival. Several “multiple-omics” studies characterizing ACC on a molecular level identified two different clusters correlating with patient survival (C1A and C1B). We here used the publicly available transcriptome data from the TCGA-ACC dataset (*n* = 79), applying machine learning (ML) methods to classify the ACC based on expression pattern in an unbiased manner. UMAP (uniform manifold approximation and projection)-based clustering resulted in two distinct groups, ACC-UMAP1 and ACC-UMAP2, that largely overlap with clusters C1B and C1A, respectively. However, subsequent use of random-forest-based learning revealed a set of new possible marker genes showing significant differential expression in the described clusters (e.g., *SOAT1*, *EIF2A1*). For validation purposes, we used a secondary dataset based on a previous study from our group, consisting of 4 normal adrenal glands and 52 benign and 7 malignant tumor samples. The results largely confirmed those obtained for the TCGA-ACC cohort. In addition, the ENSAT dataset showed a correlation between benign adrenocortical tumors and the good prognosis ACC cluster ACC-UMAP1/C1B. In conclusion, the use of ML approaches re-identified and redefined known prognostic ACC subgroups. On the other hand, the subsequent use of random-forest-based learning identified new possible prognostic marker genes for ACC.

## 1. Introduction

Adrenocortical carcinoma (ACC) is a rare endocrine malignancy with an incidence rate of approximately 0.7–2.0 per million [1] and is characterized by high aggressiveness, which leads to poor prognosis. The 5 year overall survival rate ranges from 16% to 47% and is particularly poor in patients with metastatic disease [2]. Complete surgical resection is the treatment of choice in localized ACC and is virtually the only option to achieve a cure. As recurrence is frequent, adjuvant therapy is recommended in most patients [3,4]. Despite continuous development in therapeutic concepts of ACC, the improvements brought to patient survival remain modest [5,6]. Preliminary studies on the molecular events leading to tumorigenesis in ACC [7] led to the first molecular targeted therapies, such as IGF1R (insulin-like growth factor 1 receptor) [8] and VEGF (vascular endothelial growth factor) [9] inhibitors, which all proved disappointing [10]. Given the situation only five years ago, it was even pessimistically asserted that a breakthrough might not be in sight for the next 10 to 15 years [11]. Therefore, detailed information about the molecular and genetic background of tumorigenesis in ACC is still as needed as before. In more recent years, with the advent of affordable next generation sequencing and through concerted efforts of international consortia, several pan-genomic studies were performed in adrenocortical tumors with the goal to better understand the mechanisms that lead to adrenal tumorigenesis and are linked to worse clinical outcome [12,13,14,15].

In the first integrated genomics study on ACC, Assié et al. [12] uncovered several novel molecular features by performing a multi-omics profiling of germline and somatic exomes, copy number variations, DNA methylation, as well as mRNA and miRNA expression in 45 ACC tissues. Among other things, the authors confirmed that somatic copy number alterations (gains and losses) are common in ACC as shown by prior single nucleotide polymorphism array studies [16]. While also confirming previously identified alterations in *CTNNB1*, *TP53*, *CDKN2A*, *RB1*, and *MEN1*, the authors also identified novel somatic alterations in *ZNRF3*, *DAXX*, *TERT*, and *MED12*. The gene most frequently targeted for somatic alteration was *ZNRF3*, altered in 21% of ACC and mutually exclusive with mutations in *CTNNB1*. This alteration suggests that Wnt ligands may be implicated in the tumorigenesis of a subset of ACC [17]. The authors also identified a unique miRNA signature associated with an imprinted *DLK1-MEG3* cluster downregulated in a subset of ACC that the group identified earlier and named C1B [18]. Importantly, they also showed a higher mutation rate and higher incidence of recurrent mutations in the other subset, called C1A, which was also associated with a poorer prognosis. These data were partly validated by Juhlin et al. [14], who performed whole-exome sequencing and copy number variations screening in a cohort of 41 ACC tissues.

In 2016, the largest multiplatform study on adrenocortical carcinoma to date followed as part of the consortium of genomic cancer studies—The Cancer Genome Atlas project (TCGA-ACC) [15]. The involvement of TCGA enabled the inclusion of 91 international ACC samples in the study. However, the number of samples analyzed varied for each method: whole-exome sequencing (*n* = 90), mRNA sequencing (*n* = 79), miRNA sequencing (*n* = 79), DNA copy number via SNP arrays (*n* = 89), DNA methylation via DNA methylation arrays (*n* = 79), and targeted proteome from reverse phase protein array (RPPA; *n* = 45). Compared to Assié et al., TCGA-ACC identified additional recurrent somatic alterations in *PRKAR1A*, *RPL22*, *TERF2*, and *CCNE1*, and somatic alterations in epigenetic modifiers including MLL family members, *SETD2*, *TET1*, and *SMARCA4*. Somatic mutations observed in ACC affected in ~45% of cases the cell cycle, in ~40% the Wnt pathway, and in ~20% epigenetic modifiers. Looking at the copy number alterations, TCGA-ACC identified three recurrent profiles: quiet (diploid tumor genome), chromosomal (frequent whole chromosome loss of heterozygosity and hypodiploidy in a subset of tumors), and noisy (frequent focal gains and losses). A subset of the “noisy” and “chromosomal” tumors was also characterized by whole genome doubling, associated with TERT overexpression. “Chromosomal” tumors with genome doubling and “noisy” tumors in general were also associated with worse prognosis [15].

TCGA-ACC identified that ACCs can also be classified in steroid-low/immune-high (low expression of steroidogenic markers and high-expression markers associated with an activated immune response) and steroid-high (high-expression of steroidogenic markers). Both categories can be further subdivided considering cell-cycle activation markers. Steroid-low/low proliferation tumors were associated with the previously identified “good prognosis” C1B cluster, whereas steroid-high/high-proliferation signature was associated with the “poor prognosis” C1A cluster. Combining all the data from all the different approaches, ACC-TCGA divided the ACCs into three distinct molecular subtypes, referred to as cluster of clusters (COC) 1, COC2, and COC3, directly correlating with patient prognosis: COC1 tumors—best prognosis, COC2 tumors—intermediate prognosis, and patients with COC3 tumors had the worst prognosis, with rapid disease progression [15].

What all these above-mentioned studies [13,14,15] have in common is the use of multiplatform molecular profiling and clustering of genome wide data into several prognostic relevant clusters. However, the multi-platform nature of these studies makes them also very costly and unpractical to be routinely used in patient stratification in a clinical setting. Furthermore, while defining the clustering analyses as unsupervised, the authors perform several adjustments to the datasets—for example, quantification cut-offs, selection of adrenal cortex specific markers and assisted combinations at different levels, which are introducing a scientist-biased component into the analysis, making it even harder to adapt the retrospective analysis into clinical everyday life. In this study, we present a new, simple, unsupervised, machine-learning-based method that is delivering the same clustering power for the adrenocortical tumors as the original complex analysis, based only on the mRNA expression dataset from the ACC-TCGA study and validated in a separate cohort of adrenocortical tumors that was previously evaluated by RNA-seq [19].

## 2. Materials and Methods

### 2.1. Patient Cohorts

In this work, we used the RNA-sequencing data provided by the TCGA-ACC consortium consisting of 79 ACC samples [20] (accessed on 8 August 2019). For our analyses, we used the fragments per kilobase per million (FPKM) files as input. For independent confirmation, we additionally used a dataset published recently by the ENSAT consortium [19] after being granted access to the sequencing results and clinical data. This dataset containing RNA-sequencing results, consists of ACC (*n* = 7) samples, but mainly of non-malignant forms: normal adrenal glands (NAG, *n* = 4) and adrenocortical adenomas (ACA; *n* = 52), differentiating between endocrine inactive adenomas (EIA; ns = 9), adenomas with mild autonomous cortisol secretion (MACS-CPA; *n* = 17) and Cushing syndrome cortisol producing adenomas (CS-CPA; *n* = 26). As this study is only an in silico reanalysis of previously published data, no ethic committee approval was needed.

### 2.2. Bioinformatics Analyses

A Jupyter Notebook environment (version 7.5.0) was used to perform all bioinformatic steps using Python version 3.6.9, scikit-learn version 0.22.1 [20], SciPy version 1.3.0 [21] and pandas version 0.24.2 [21,22]. The notebook for the unsupervised UMAP clustering is available upon request.

#### 2.2.1. Uniform Manifold Approximation and Projection (UMAP) Clustering

For UMAP clustering and plotting, we used euclidean_distances from the sklearn.metrics.pairwise module to determine the squared pairwise Euclidean distance between samples of the initial data set, on which the local connectivity parameter rho, together with the first nearest neighbor, is based. For each entry of the distance matrix, the sum of probabilities in the high-dimensional space is calculated. The nearest neighbors and the probabilities for each entry determine the entropy and, based on a binary search the optimal rho for a fixed number of the 15 nearest neighbors is computed. To satisfy the symmetry condition of the UMAP algorithm we used a simplified calculation: instead of subtracting the product of the probability and the transposed probability from the sum of the probability and the transposed probability, we divided the sum by 2. For the subsequent building of low-dimensional probabilities we used mind_dist = 0.25. As a cost function, we used cross-entropy—with a normalized Q parameter. The gradient of it was used in the gradient descent learning—using the regular instead of the stochastic one with 2 dimensions and 50 neighbors.

Based on the results of the UMAP, we manually curated the data, determined the clusters for subsequent analysis and deleted three outliers (TCGA-OR-A5J8, TCGA-OR-A5JB, and TCGA-P6-A5OG—Table S1). Two of these three outliers (TCGA-OR-A5J8 and TCGA-OR-A5JB) have been classified as sarcomatoid samples in the original publication and were, therefore, expected to be outliers. The last datapoint (TCGA-P6-A5OG) was not described at all in the original work but, as all three samples cluster closely together, is most probably also a sarcomatoid sample. We then again performed the described UMAP plotting with the curated data for better cluster representation, obtaining two distinct clusters, which we named ACC-UMAP1 and ACC-UMAP2 according to their position in the given UMAP.

#### 2.2.2. Random Forest Learning

Based on the obtained clusters, we trained a supervised random forest (RF) classifier (RandomForestClassifier of the sklearn.ensemble module) to specify the transcriptional differences—based on unprocessed FPKM values—between the two identified clusters. For training our model, we used a 50/50 split, letting the model learn on 50% of the data and evaluating it on the other 50%, with 1000 trees in the forest (n_estimators = 1000). We trained 100 models and determined the 100 features—representing the ensemble gene IDs—with the highest impact on the model using the according “feature values”, which imply the importance of the corresponding feature. For each feature, we counted its occurrence in the top 100 for each of the 100 trained models, creating a form of ensemble technique. For subsequent analysis, the combined top 100 genes—according to the number of appearances in the top 100 of each individual model and the calculated mean rank—from these, 100 different models were used, adapted from a previous analysis [23]. For the 100 trained models, the minimum testing accuracy is 81.58%, the maximum testing accuracy is 100%, and the mean testing accuracy over all different models is 95.5%. Within these 100 trained models, 18 had a testing accuracy of 100%. The 5-fold cross-fold validation yielded a mean accuracy of 96.00% ± 5.33%.

#### 2.2.3. Mutation Analysis

For further insight into the differences between the determined clusters, we also investigated common mutations for ACC, namely *TP53*, *CTNNB1*, *NF1*, *APC*, *ZNRF3*, *MEN1*, *GNAS*, and *ATRX*. The information on the mutational status of the samples were obtained from cbioportal (https://www.cbioportal.org/ accessed on 2 September 2020) [24,25].

#### 2.2.4. Plots and Statistical Analysis

Box and scatter plots were generated using matplotlib. For survival analysis, Kaplan Meier (KM) plots were generated using the lifelines module (version 0.23.1) [26]. If not stated otherwise, the statistical tests for clinical characteristics and mutation analysis were performed using Kruskal–Wallis-Test—using scipy.stats module including indicated significances in the box and scatter plots, for which we used the statannot module for python (version 0.2.2). For the analysis of further interactions and relations between the identified top 100 genes, we used a network generated by StringDB [27] showing a close relation of the genes used for further analysis. Kaplan–Meier followed by Cox regression analysis was used to estimate overall survival (OS) using IBM SPSS v 26 for Windows.

## 3. Results

### 3.1. An UMAP Clustering Approach Is Able to Generate Two Distinct Clusters of ACC Samples That Largely Confirm Previously Published Clusters and Correlate with Patient Survival

**Figure 1:**
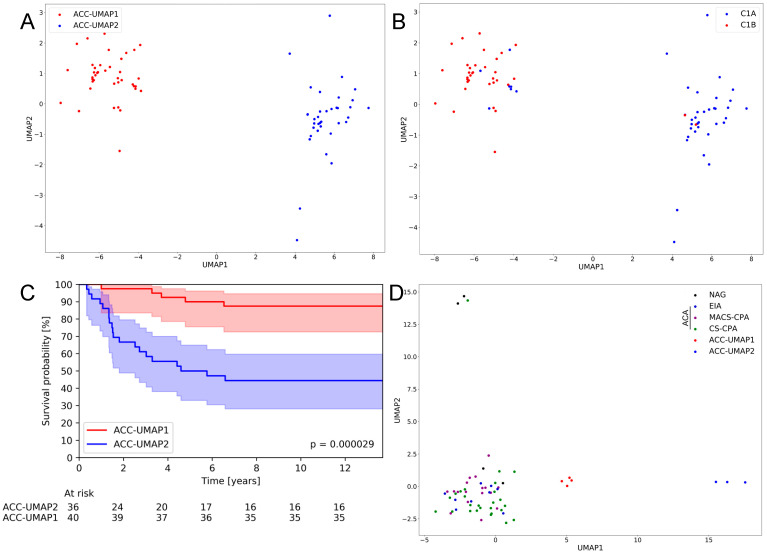
UMAP cluster representation of different mRNA expression patterns. Representation of the UMAP (uniform manifold approximation and projection) clustering of the ACC-TCGA dataset without outliers (**A**) and the overlap with C1A/C1B clustering from the original publication of Zheng et al. [15] (**B**). Kaplan–Meier curve of overall survival of ACC-TCGA patients assigned to the two clusters by UMAP. Shaded area: confidence interval with alpha = 0.05 (**C**). Representation of the UMAP clustering of the ENSAT dataset [19] (**D**). NAG = normal adrenal gland; EIA = endocrine inactive adenoma; MACS-CPA = mild autonomous cortisol secretion adenoma-cortisol producing adenoma; CS-CPA = Cushing syndrome-cortisol producing adenoma which together make the ACA = adrenocortical adenoma; ACC = adrenocortical carcinoma.

In a first UMAP clustering approach of the log transformed FPKM values of the whole TCGA-ACC dataset, most of the samples were attributed to two large clusters, with only three samples not fitting in these clusters (Figure S1A). After curating the dataset by eliminating these outliers from the analysis (see Table S1), the subsequent UMAP provided two distinct clusters, which we named “ACC-UMAP1” (the left cluster) and “ACC-UMAP2” (the right cluster) (Figure 1A). We correlated the samples from these two clusters with the different clustering characteristics that were attributed to these samples in the original description by Zheng et al. [15] and, interestingly, the clusters generated by our UMAP approach overlapped very well with several clusters published before (Table S1). Most importantly, the clusters identified this way overlapped nearly completely with the clusters C1A and C1B from the Zheng et al. study (Figure 1B), with only 9 samples (11.84%) not directly matching our cluster assignment. As clusters C1A and C1B were already shown to tightly correlate with patient prognosis [12], it was no surprise that the two ACC-UMAP clusters also correlated very well with the overall survival of the patients (12.46 (95%CI 11.43–13.48) vs. 7.38 (95%CI 5.48–9.27) years, hazard ratio for death 6.27 (95%CI 2.34–16.77, *p* = 0.000029) (Figure 1C). Cluster ACC-UMAP1, mostly overlapping with the C1B cluster, is associated with a better prognosis, while ACC-UMAP2 is associated with a poorer prognosis, as previously described. The ACC-UMAP clusters also correlated very well with other clusters from Zheng et al., like the steroid and immune phenotype with only 11 samples (14.47%) off (Figure S1B), and with the COC with only 9 samples (11.84%) that clustered differently (Figure S1D). In contrast, the genomic doubling clusters from Zheng et al. were distributed independently over the two described UMAP clusters (Figure S1C).

We applied the same UMAP approach to a dataset published recently by the ENSAT consortium [19] which contained only 7 ACCs but many other adrenocortical tissues, either from normal adrenal glands or from different benign adrenocortical tumors, as previously described [19]. Interestingly, the obtained clusters for the ACC samples show a similar clustering to the ACC-TCGA samples, with an ACC-UMAP1 cluster on the left side and an ACC-UMAP2 on the right side, even though the sample numbers are comparatively low (Figure 1D). Additionally, the number of samples per cluster with roughly 50% each (4 left, 3 right) is comparable to the ACC-TCGA results (40 left, 36 right). Due to the low number of ACC samples in this dataset, we could not perform a statistically relevant analysis regarding patient survival, however, we observed that 2 out 3 (66.7%) ACC of the ACC-UMAP2 cluster died (median survival was 7.25 years), whereas none of the ACC patients of the ACC-UMAP1 cluster died due to the disease during the time interval of the study. Another interesting cluster is the one containing nearly all of the benign tumor samples, which is close to the ACC-UMAP1 cluster, showing a closer relation between these two (Figure 1D).

### 3.2. Random Forest Analysis Identifies 100 Genes That Are Differentially Expressed in Cluster ACC-UMAP2, but Most of These Genes Have Not Yet Been Associated with Adrenocortical Tumorigenesis

**Figure 2:**
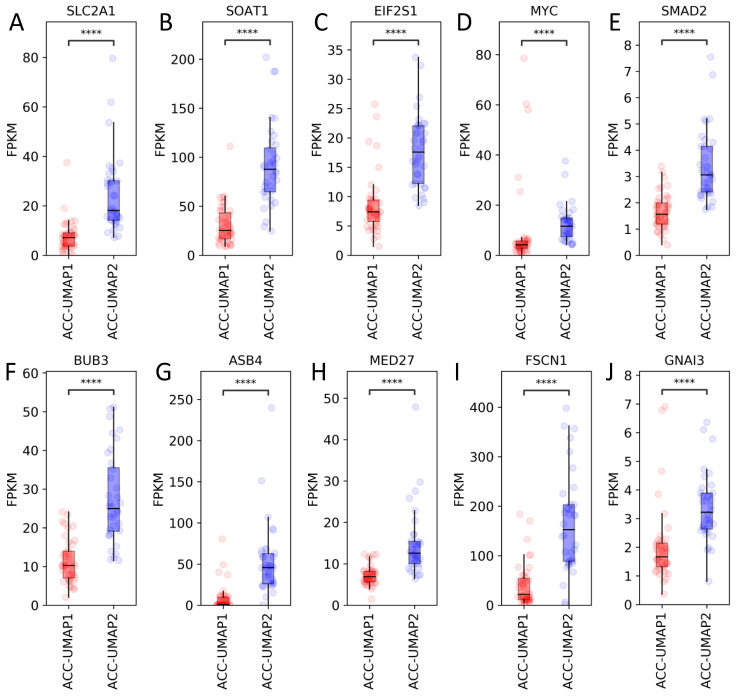
Selection of mRNA expression pattern of 10 genes from the TCGA-ACC dataset, as identified by RF analysis, that were previously shown to be involved in adrenal function. *SLC2A1*: solute carrier family 2 member 1 (**A**), *SOAT1*: sterol-O acyltransferase (**B**), *EIF2S1*: eukaryotic translation initiation factor 2 α (**C**), *MYC* proto-oncogene MYC (**D**), *SMAD2*: sma—and mad-related protein 2 (**E**), *BUB3* budding uninhibited by benzimidazoles 3 homolog (**F**), *ASB4*: ankyrin repeat And SOCS box containing 4 (**G**), *MED27*: mediator complex subunit 27 (**H**), *FSCN1*: fascin actin-bundling protein 1 (**I**), *GNAI3*: guanine nucleotide binding protein (G protein), alpha inhibiting activity polypeptide 3 (**J**). ns, not significant. **** *p* < 0.0001. *Y*-axis units: FPKM.

Being able to recreate already established ACC clusters with our UMAP approach, we were interested in the molecular differences between the identified clusters. Applying RF learning, we were able to determine the 100 genes with the most influence in distinguishing our clusters (Figure S2). Further analyses revealed that 98 of these 100 genes were overexpressed in the ACC-UMAP2 cluster as compared to the ACC-UMAP1 cluster of the ACC-TCGA data (Figure 2, Table S2). The only two exceptions were *CSGALNACT1*, encoding for chondroitin sulfate N-acetylgalactosaminyltransferase 1, an enzyme usually associated with cartilage development and *KLRB1*, encoding for the killer-cell lectin-like receptor B1, a type II membrane protein known to play an inhibitory role on natural killer cell cytotoxicity (Figure S2). Surprisingly, the vast majority of the 100 genes identified by the RF analysis have little known connection with the adrenocortical function and tumorigenesis. Notably, among the known genes we found the solute carrier family 2 member 1/glucose transporter 1 (*SLC2A1/GLUT1*) (Figure 2A), an important, stage independent predictor of ACC patient outcome [28] as well as those encoding for the sterol-O acyltransferase (*SOAT1*) (Figure 2B) and eukaryotic translation initiation factor 2 α (*EIF2S1*) (Figure 2C), both known to be involved in endoplasmic reticulum stress processes in the adrenocortical tissues associated with mitotane treatment and also having an influence on ACC patient outcome [29,30]. There were also other interesting genes overexpressed in the poor survival cluster ACC-UMAP2 that were already reported in the context of adrenal function disturbances, such as the proto-oncogene *MYC* (Figure 2D) [31] the TGF-β signal transducer *SMAD2* (Sma—and mad-related protein 2) (Figure 2E) [32], the mitotic checkpoint gene *BUB3* (udding uninhibited by benzimidazoles 3 homolog) (Figure 2F) [33] and *ASB4* (ankyrin repeat and SOCS box containing 4) (Figure 2G) [20]. *MED27* (mediator complex subunit 27) (Figure 2H), a cofactor involved in the transcriptional initiation by the RNA polymerase II apparatus was shown to be involved in adrenal cortical carcinogenesis by targeting the Wnt/β-catenin signaling pathway and the epithelial-mesenchymal transition process [34]. *FSCN1* (Figure 2I), a fascin family member, was recently shown to be associated with tumor invasiveness in ACC [35] and *GNAI3* (guanine nucleotide binding protein (G protein), alpha inhibiting activity polypeptide 3) (Figure 2J) was shown to be increased in nutrient starved adrenal glands in RGS4_ko_ mice [36].

**Figure 3:**
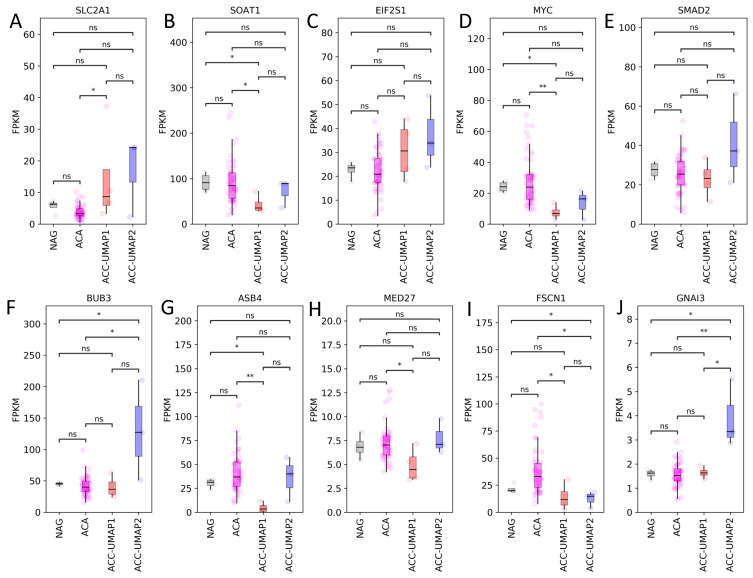
Selection of mRNA expression pattern of 10 genes from the validation dataset, as identified by RF analysis, that were previously shown to be involved in adrenal function. *SLC2A1*: solute carrier family 2 member 1 (**A**), *SOAT1*: sterol-O acyltransferase (**B**), *EIF2S1*: eukaryotic translation initiation factor 2 α (**C**), *MYC*: proto-oncogene MYC (**D**), *SMAD2*: sma—and mad-related protein 2 (**E**), *BUB3*: budding uninhibited by benzimidazoles 3 homolog (**F**), *ASB4*: ankyrin repeat and SOCS box containing 4 (**G**), *MED27*: mediator complex subunit 27 (**H**), *FSCN1*: fascin actin-bundling protein 1 (**I**), *GNAI3*: guanine nucleotide binding protein (G protein), alpha inhibiting activity polypeptide 3 (**J**). NAG = normal adrenal gland; ACA = adrenocortical adenoma; ACC = adrenocortical carcinoma. ns, not significant. * *p* < 0.05, ** *p* < 0.01, ns, not significant. *Y*-axis units: FPKM.

Looking at the mRNA expression of the same factors in the validation dataset, it became obvious that, while some of the genes followed the same pattern of expression as in the ACC-TCGA dataset, some differed (Figure S3A). Furthermore, in the validation cohort we observed only a tendency of overexpression in most of the genes, without significant differences (Table S3), probably due to the low number of ACC cases in this dataset. However, more interesting are the differences in expression between the two ACC clusters and the normal adrenal glands and adrenocortical adenomas (Figure S3A and Figure 3). For example, while the expression of *SLC2A1* (GLUT-1) is higher in ACC than in NAG and adenomas and highest in the ACC-UMAP2 cluster (Figure 3A), the expression of *MYC* for example is significantly lower in both ACC clusters when compared to the NAG (Figure 3D). This is in conformity with previously published data that shows low *MYC* expression in adrenocortical tumors [31,37]. We performed these analyses while also considering the different ACA entities (EIA, MACS-CPA and CS-CPA) separately (Figure S3B, Table S4), however, as there were no significant differences between the three subgroups, we pooled all ACAs together for the main analysis.

To gain further insight into possible connections of the identified genes, we performed a network analysis, showing that overall, half of the top 100 genes is interconnected in a large network that is involved in both cell division and transcription control.

### 3.3. Mutational Analysis Reveals CTNNB1 and TP53 as the Only Known Differentially Mutated Genes

Previous studies have already shown the close relation between the C1A/C1B clusters and mutation status. To further confirm our used approach, we additionally looked at driver mutations and their impact on cluster affiliation. Analysis of known driver mutations in ACC, including TP53, CTNNB1, NF1, APC, ZNRF3, MEN1, GNAS, and ATRX, show that there is only a small proportion of genes that are significantly altered within the UMAP identified clusters. For NF1, APC, ZNRF3, GNAS, and ATRX, no significance could be observed. Only for the genes TP53 (ACC-UMAP2 vs. ACC-UMAP1: 11 vs. 1 sample, *p* = 0.042) and CTNNB1 (ACC-UMAP2 vs. ACC-UMAP1: 12 vs. 1 sample, *p* = 0.00026) were significant results present with a higher proportion of mutated samples in the right cluster. For MEN1 a tendency was observable (*p* = 0.058), also with more mutated samples in the right cluster. As such, these analyses confirm our used approach and confirm the cluster ACC-UMAP2 as the worse cluster regarding both survival and distribution of mutated genes.

## 4. Discussion

In comparison to Zheng et al. [15], our approach considers only the mRNA expression, as it was performed previously by de Reyniès et al., in 2009 [18]. At that time, a gene signature was determined on the basis of mRNA from microarrays, based on hierarchical clustering methods [7], which identified the two groups C1A/C1B. Compared to Zheng et al., who performed a pre-selection of genes before the clustering analysis (only considering the genes that are expressed in more than 25% of the samples and then only the 5000 most variable genes), we do not limit the amount of data in our approach using all possible 60.483 transcripts provided by TCGA for our analysis, which is also the strength of our study. Despite this difference, we can almost completely confirm the grouping according to C1A and C1B, just as Zheng et al. had in their “K2” approach, who already showed the separability into these two groups in their data. When we split the clusters further to take into consideration the samples that clustered differently between the C1A/B system and our ACC-UMAP1/2 system, it became clear that the unbiased UMAP clustering system is more robust in clustering together samples with similar expression patterns. This is shown by the fact that in the majority of the split cases the differences between the different UMAP sub-clusters were non-significant while this was not the case for the C1A/B sub-clusters.

The subsequent use of a RF to identify the transcriptomic differences between the two groups shows great differences between the two approaches. While Zheng et al. name 151 genes in their K4 approach and de Reyniès et al. can limit their overall survival prediction to only 2 genes (*BUB1B* and *PINK1*) [18], we show 100 genes that are most likely to separate the two clusters found. Because of the unbiased consideration of all possible transcripts, it is not surprising that the top 100 genes identified are mostly unknown in the field of tumorigenesis or adrenocortical function, because preselection of variable genes was widely used before the era of machine learning. This might be considered a weakness of our method and leads to apparently strange results. For example, the overlap of the top 100 genes of our approach compared to the K4 approach of Zheng et al. is just one gene—*ASB4*. It is also striking that 98 genes are overexpressed in one of the clusters and only two in the other cluster. However, this is solely a representation of the approach, which represents the most influential genes for the learned models. Despite this, we could still find at least 10 genes among the top 100 that were previously reported in the context of the adrenal function, underlining their importance in the adrenocortical disease progression, also strengthening the results and the approach. The prognostic role of some of these genes was reported before, as in the case of *SLC2A1/GLUT1* [28] or *FSCN1* [35]. In other cases, such as with *GNAI3*, an increase in gene expression was reported in nutrient starved adrenal glands in a mouse model [36], starvation that is often observed in adrenal cancer. The fact that GNAI3 expression is highest in the poor prognosis cluster ACC-UMAP2 and low in ACC-UMAP1 and benign adrenal tissues show that this gene has high prognostic potential. Nevertheless, a gene does not have to already be reported in the context of adrenal disease to be considered a good prognostic candidate. Just to take one example, while not yet analyzed in adrenal cancer, *CBX3* (Chrombox 3) has a similar expression pattern between the different clusters with low expression in normal and benign adrenocortical tissues and high expression in ACC, especially in the poor prognosis cluster ACC-UMAP2. While it has no obvious connection with adrenal function, it is a gene that is involved in histone methylation and was associated with other types of cancer [38]. We are confident that the future analysis of the RF generated top 100 list of genes will bring to light several new prognostic markers for ACC.

Our results also show that for the known mutations, *CTNNB1* and *TP53* both cluster significantly differently between the two ACC-UMAP clusters. Combining these results with the tendency observed for *MEN1* (1 mutated sample in the left and 5 in the right cluster) and the significant survival differences between the two clusters, it can be indirectly assumed that these mutations do have an impact on patient survival.

Here we show a novel, completely unbiased way to clusters the TCGA-ACC dataset without limiting the input data. We were able to clarify and maybe even refine the already established and well-known ACC subgroups C1A and C1B described by TCGA-ACC. Also, the novel differentially expressed genes discovered by our approach should be further investigated and verified in future work regarding their potential role as prognostic biomarkers.

## 5. Conclusions

In the present work, we applied machine-learning methods to a published ACC dataset generated by the TCGA consortium and validated it in an ENSAT generated dataset. First, we applied UMAP, a standard clustering method in single-cell sequencing analysis, to identify possible clusters within the data. This approach yielded two clusters that match to a large extent (>80%) the already published and well-known ACC clusters (C1A/C1B). Subsequent survival analyses confirmed the clusters found by our approach and show a significant survival advantage for the ACC-UMAP1 cluster (corresponding to the already described C1A cluster). Examination of known mutations distribution within the clusters showed a significant accumulation of mutations of the *CTNNB1* and *TP53* genes in the poorer survival cluster (ACC-UMAP2). The subsequent use of a RF learning revealed the 100 genes that have the greatest influence on the separation of the two clusters and could potentially serve as new biomarkers or novel targets for therapeutic approaches. Taken together, we were able to show the capabilities of machine-learning-based methods by identifying and redefining the already well-known C1A and C1B cluster of the TCGA-ACC cohort and opening up their further evaluation and use in sub-group identification research also for other entities.

## Data Availability

The results shown here are based upon data generated by the TCGA Research Network: https://www.cancer.gov/tcga (accessed on 8 August 2019) and the European Network for the Study of Adrenal Tumors/ENSAT (ensat.org, accessed on 8 August 2019). The original data can be explored through the Broad Institute GDAC FireBrowse portal (http://firebrowse.org/?cohort=ACC, accessed on 8 August 2019) and the platform EGA (https://www.ebi.ac.uk/ega/home, accession number EGAS00001004533), respectively.

## Supplementary Materials

The following are available online at https://www.mdpi.com/article/10.3390/cancers13184671/s1, Table S1. Clinical characteristics and different associated clustering of the ACC samples in the ACC-TCGA cohort; Table S2. Differential mRNA expression levels of the 100 genes selected by random forest in the ACC-TCG) cohort; Table S3. Differential mRNA expression levels of the 100 genes selected by random forest in the validation (ENSAT) cohort considering all the ACA subgroups together; Table S4. Differential mRNA expression levels of the 100 genes selected by random forest in the validation (ENSAT) cohort considering all the ACA subgroups separately; Figure S1. Various UMAP (Uniform Manifold Approximation and Projection) cluster representations for the TCGA-ACC dataset. Representation of the UMAP of the. (A) ACC-TCCA dataset and the overlap with different molecular clustering from the original publication of Zheng et al. [15] without outliers: steroid phenotype (B), genome doubling (gd; (C)) and cluster of clusters (COC; (D)).; Figure S2. mRNA expression pattern of all top 100 genes, in alphabetical order, from the validation dataset, considering the different ACA entities separately, as identified by random forest. NAG = normal adrenal gland; EIA = endocrine inactive adrenocortical adenoma; MACS-CPA = mild autonomous cortisol secreting adrenocortical adenoma; CS-CPA = Cushing syndrome cortisol producing adenoma; ACC = adrenocortical carcinoma. ns, not significant. *p* < 0.05, * *p* < 0.01, *** *p* < 0.0001. Y-axis units: FPKM; Figure S3. STRING-DB network (https://string-db.org/, accessed on 8 August 2019) analysis of known interactions between the top 100 genes as identified by random forest learning to separate the ACC-TCGA dataset in clusters.
