# Supplementary figures and images for: Identifying New Potential Biomarkers in Adrenocortical Tumors Based on mRNA Expression Data Using Machine Learning
Source: Cancers (Basel). 2021 Sep 17;13(18):4671. doi: 10.3390/cancers13184671 (PMC8469239; doi:10.3390/cancers13184671)

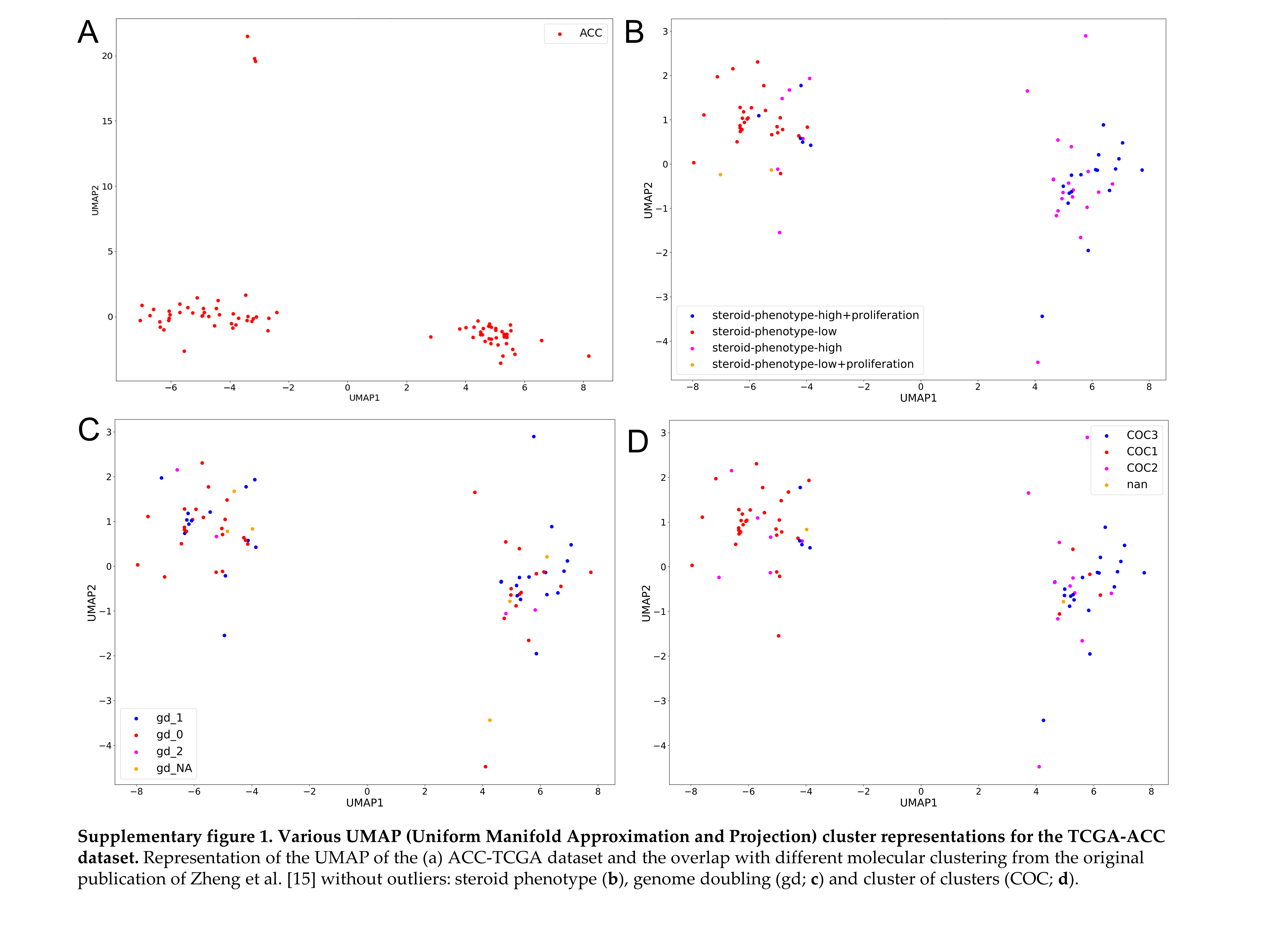

Supplement: Supplementary file 1 [file cancers-13-04671-s001.zip › cancers-1325927-Supplementary/Supplementary figure 1 rev.png]

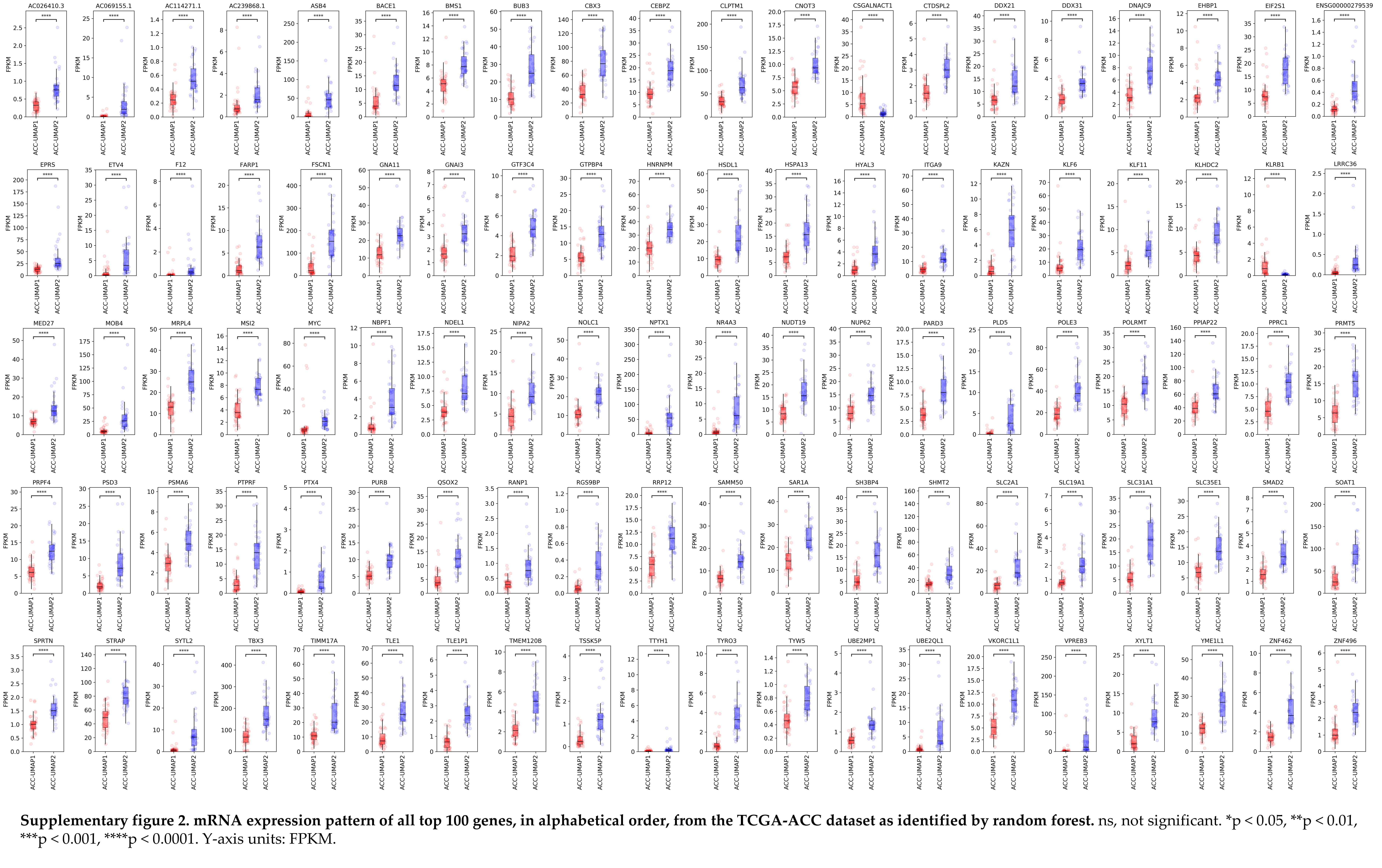

Supplement: Supplementary file 1 [file cancers-13-04671-s001.zip › cancers-1325927-Supplementary/Supplementary figure 2 rev.png]

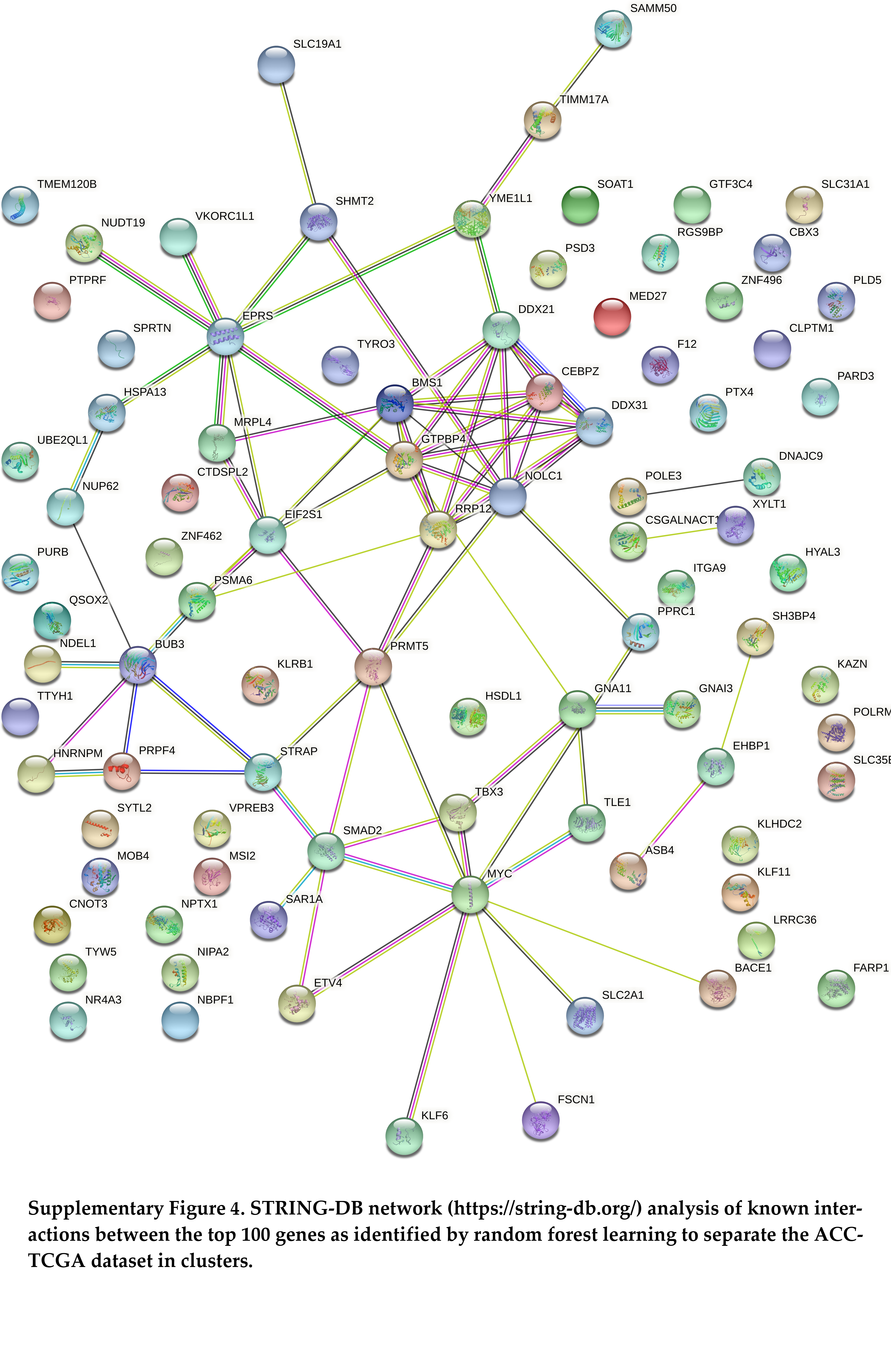

Supplement: Supplementary file 1 [file cancers-13-04671-s001.zip › cancers-1325927-Supplementary/Supplementary figure 4 rev.png]
